# Mitigation of Salinity-Induced Oxidative Damage, Growth, and Yield Reduction in Fine Rice by Sugarcane Press Mud Application

**DOI:** 10.3389/fpls.2022.840900

**Published:** 2022-04-26

**Authors:** Imran Khan, Awon Muhammad, Muhammad Umer Chattha, Milan Skalicky, Muhammad Bilal Chattha, Muhammad Ahsin Ayub, Muhammad Rizwan Anwar, Walid Soufan, Muhammad Umair Hassan, Md Atikur Rahman, Marian Brestic, Marek Zivcak, Ayman El Sabagh

**Affiliations:** ^1^Department of Agronomy, University of Agriculture, Faisalabad, Pakistan; ^2^Department of Botany and Plant Physiology, Faculty of Agrobiology, Food, and Natural Resources, Czech University of Life Sciences Prague, Prague, Czechia; ^3^Department of Agronomy, Faculty of Agricultural Sciences, University of the Punjab, Lahore, Pakistan; ^4^Rice Research Station, Bahawalnagar, Pakistan; ^5^Plant Production Department, College of Food and Agriculture Sciences, King Saud University, Riyadh, Saudi Arabia; ^6^Research Center on Ecological Sciences, Jiangxi Agricultural University, Nanchang, China; ^7^Grassland and Forage Division, National Institute of Animal Science, Rural Development Administration, Cheonan-si, South Korea; ^8^Laboratory Slovak University of Agriculture in Nitradisabled, Nitra, Slovakia; ^9^Institut of Plant and Environmental Sciences, Faculty of Agrobiology and Food Resources, Slovak University of Agriculture, Nitra, Slovakia; ^10^Department of Agronomy, Faculty of Agriculture, Kafrelsheikh University, Kafrelsheikh, Egypt

**Keywords:** anti-oxidants, ionic balance, photosynthetic pigments, press mud, rice, salt stress

## Abstract

Salinity stress is one of the major global problems that negatively affect crop growth and productivity. Therefore, ecofriendly and sustainable strategies for mitigating salinity stress in agricultural production and global food security are highly demandable. Sugarcane press mud (PM) is an excellent source of the organic amendment, and the role of PM in mitigating salinity stress is not well understood. Therefore, this study was aimed to investigate how the PM mitigates salinity stress through the regulation of rice growth, yield, physiological properties, and antioxidant enzyme activities in fine rice grown under different salinity stress conditions. In this study, different levels of salinity (6 and 12 dS m^–1^) with or without different levels of 3, 6, and 9% of SPM, respectively were tested. Salinity stress significantly increased malondialdehyde (MDA, 38%), hydrogen peroxide (H_2_O_2_, 74.39%), Na^+^ (61.5%), electrolyte leakage (40.32%), decreased chlorophyll content (32.64%), leaf water content (107.77%), total soluble protein (TSP, 72.28%), and free amino acids (FAA, 75.27%). However, these negative effects of salinity stress were reversed mainly in rice plants after PM application. PM application (9%) remained the most effective and significantly increased growth, yield, TSP, FAA, accumulation of soluble sugars, proline, K^+^, and activity of antioxidant enzymes, namely, ascorbate peroxidase (APX), catalase (CAT), and peroxidase (POD). Thus, these findings suggest a PM-mediated eco-friendly strategy for salinity alleviation in agricultural soil could be useful for plant growth and productivity in saline soils.

## Introduction

Salinity stress is one of the serious limitations for plant growth, productivity, and soil health (Machado and Serralheiro, 2017; Kamran et al., 2020; Seleiman et al., 2022). It is expected that salinity contamination will severely affect 50% of the total cultivable land within 2050 (Shrivastava and Kumar, 2015). Salinity negatively affects crop production and causes huge yield and economic losses; therefore, proper strategies must be adopted to reduce salinity stress on crops and protect the soils from the devastating impacts of salinity stress (Mohanavelu et al., 2021; Monsur et al., 2022). Salinity stress is a complex process that negatively affects the plant’s physiological and biochemical processes (Dustgeer et al., 2021; Sultan et al., 2021). The effects of salt ranged from seed germination to flowering and fruiting settings, which resultantly caused significant yield losses (Mbarki et al., 2020; El Sabagh et al., 2021). Moreover, salt stress also induces osmotic and ionic stress and induce the production of reactive oxygen species (ROS), which cause damages to significant molecules, namely, DNA, proteins, and cellular membranes (Ahanger et al., 2017; Hassan et al., 2019, 2020, 2021a; Mbarki et al., 2020; Hossain et al., 2021; Sultan et al., 2021; Batool et al., 2022). Salt stress also reduced the nutrient uptake and enzymes’ activity, therefore significant growth and yield losses (Kamran et al., 2020; Singhal et al., 2021). Moreover, salinity stress also reduced the photosynthetic efficiency and increased the accumulation of toxic ions (Na^+^), which cause serious growth and yield losses (Ahanger et al., 2017; Mbarki et al., 2020; Taha et al., 2020; Dustgeer et al., 2021; El Sabagh et al., 2021; Sultan et al., 2021). The adverse effects of salt stress on plants growth and development depend on different factors, namely, planting species, growth stage, environmental conditions, and salts concentration in soil solution (Aghighi et al., 2018; Monsur et al., 2020). Therefore, it is essential to find out sustainable strategies to reduce the deleterious impacts of salt stress through genetic techniques, soil conditioners, and biological products (Farid et al., 2020; Leal et al., 2020; Naz et al., 2021).

For salt-affected reclamation, applying organic amendments like farmyard manures, press mud (PM), and green manuring is considered a simple and practical economic approach (Hassan et al., 2021b). Press mud is a by-product of the sugarcane industry produced in large quantities. The agricultural use of PM has shown significant improvement in nutrient uptake and soil health (Chattha et al., 2019). Sugarcane PM is considered to positively affect the soil structure, soil organic matter, nutrients uptake, and soil microbial activities (Nawaz et al., 2017; Chattha et al., 2019). Therefore, PM could serve as an important amendment to improve salt-affected soils’ productivity in this context. PM application induced salinity tolerance by enhancing the leaf water status, membrane stability, photosynthetic efficiency, accumulation of osmolytes, and K^+^ (Sheoran et al., 2021a). Press mud application improves nutrient uptake and significantly increases growth and biomass productivity under salinity stress (Imran et al., 2021). Moreover, PM also mobilizes the CaCO_3_ to maintain the optimum Ca^2+^ availability, improve soil structure stability, and increase the leaching of Na^+^ in salt-affected soils (Muhammad et al., 2019). In addition, PM also serves as a potential nutrient source and soil conditioner for reclamation of salt-affected soils (Kumar S. et al., 2017).

Rice (*Oryza sativa* L.) is an imperious staple food crop around the globe; however, salinity stress is a significant threat to rice productivity. Rice plants alleviate the salinity-induced damages by maintaining ionic balance, osmotic adjustments, scavenging ROS, maintaining nutrient uptake and cell signaling, and increasing hormonal accumulation (Liu et al., 2021). However, limited studies are conducted to determine the impact of PM on crops grown under salt stress conditions. Therefore, more investigations are required to explore the mechanism behind PM-induced salinity tolerance in field crops. To our best knowledge, no study is available related to the effect of sugarcane PM on photosynthetic pigments, physiological attributes, element concentration, antioxidant defense, and growth and yield of rice crops growing in salt-stressed conditions. Thus, we hypothesized that the application of PM would improve salinity tolerance in rice crops by favoring photosynthetic pigments, physiological attributes, and antioxidant defense. Therefore, this study was aimed to explore the impact of PM photosynthetic pigments, physiological features, H_2_O_2_ and malondialdehyde (MDA) production, soluble protein, free amino acid and elements accumulation, antioxidant defense, and growth and yield of rice crop.

## Materials and Methods

### Experimental Details

This experiment was carried out at the Agronomy Farm in a complete randomized design (CRD) with a factorial combination having three replications. The investigation was composed of several salinity treatments; control, 6 and 12 dS m^–1^, and diverse levels of PM control, 3, 6, and 9%. For achieving the desired rates of PM (3, 6, and 9%), we used PM at the rate of 300, 600, and 900 g/pot. The PM was thoroughly mixed in soil and left for 7 days. The rice variety Kisan-basmati was used in this study, which is sensitive against salinity stress. Rice plants were grown at Agronomy Farm for 4 weeks to reach the transplanting stage; after that, they were transplanted to experimental pots with a volume of 8 kg and diameter of 32 cm. In each pot, 8 kg of soil was filled and eight seedlings were transplanted in each pot. The soil used in the study had a sandy loam texture with pH (7.82), organic matter (0.82%), and available nitrogen (N), phosphorus (P), and potassium (K): 0.04%, 6.60 and 156 ppm, respectively. Ammonium phosphate (5.58 g) and sulfate of potash (1.87 g) were added to each plot to fulfill nutrient requirements. The fertilizers were applied for soil analysis, and these fertilizers were readily available in the market; therefore, they were used to meet nutrient needs. According to treatments, the imposition of various salt stress levels was applied following the procedure used previously by Khaliq et al. (2015). A soil sample from the collected soil was taken, soil paste was made, and then left for 2 h to attain equilibrium. The soil extract was then collected using filter paper. After that, the soil mixture was ovendried, and the percentage of soil saturation was calculated using the following equation.


$$Saturation\left(\%\right)=\frac{L\,o\,s\,s\,i\,n\,s\,o\,i\,l\,w\,e\,i\,g\,h\,t\,o\,n\,d\,r\,y\,i\,n\,g}{W\,e\,i\,g\,h\,t\,o\,f\,s\,o\,i\,l\,a\,f\,t\,e\,r\,d\,r\,y\,i\,n\,g}\times 100$$

The concentration of salts (6 and 12 dS m^–1^) to achieve the desired treatment levels treatments was calculated using the following equation (Khaliq et al., 2015).


$$N\,a\,c\,l\,r\,e\,q\,u\,i\,r\,e\,d\,\left(\frac{g}{k\,g}\right)=\frac{TSS\times 58.5\times saturation\left(\%\right)}{100\times 1000}$$

Here TSS refers to total soluble salt, which was determined as (EC_2_ – EC_1_) × 10. EC_1_ refers to the initial EC of soil, whereas EC_2_ refers to the desired EC as per treatments.

### Growth Parameters

Three plants were uprooted carefully from each pot, and roots and shoots were separated and washed to remove any debris. After that, the length of three roots and shoots was measured, and the average was taken. Later on, they were weighed to determine the fresh weight and ovendried (70°C) for 8 h to determine the dry weights. Moreover, three plants in each pot were selected, leaves were counted, and the average was worked out. The growth traits were collected at the flag leaf stage of the plant. The pots were well irrigated after 24 h. Plants were carefully uprooted from the pots to avoid any damage to roots.

### Determination of Photosynthetic Pigments

Leaves were selected at the flag leaf stage to determine the concentrations of chlorophyll and carotenoid by the methods of Lichtenthaler (1987). The leaves rice (1 g) was taken and homogenized in 85% of acetone solution, and the extract was centrifuged for 15 min at 1,000 rpm. Absorbance was recorded using a spectrophotometer, and the concentration of photosynthetic pigments was determined using the below equations.


$$c\,h\,l\,o\,r\,p\,h\,y\,l\,l\,a\,\left(\frac{m\,g}{g}\,F\,W\right)$$

$$\;=\frac{12.7\,\left(O\,D\,663\right)-2.69\,\left(O\,D\,645\right)\times V\times W}{1000}.$$

$$c\,h\,l\,o\,r\,p\,h\,y\,l\,l\,\,\,b\,\left(\frac{m\,g}{g}\,F\,W\right)$$

$$\;=\frac{12.9\,\left(O\,D\,645\right)-4.68\,\left(O\,D\,663\right)\times V\times W}{1000}$$

$$T\,o\,t\,a\,l\,\,\,c\,h\,l\,o\,r\,p\,h\,y\,l\,l\,\,\left(\frac{m\,g}{g}\,F\,W\right)$$

$$\;=\frac{2.02\,\left(O\,D\,645\right)-8.02\,\left(O\,D\,663\right)\times V\times W}{1000}$$

In the above equations, *V* and *W* refer to the volume of acetone and the weight of the plant sample. Moreover, carotenoids were calculated using the following equation.


$$C\,a\,r\,o\,t\,e\,n\,o\,i\,d\,s\,\left(\frac{m\,g}{g}\,F\,W\right)$$

 =O⁢D⁢480+(0.114×O⁢D⁢663)-(0.638×O⁢D⁢645)

### Measurement of Relative Water Content and Electrolyte Leakage

For the determination of RCW, leaf samples were collected at the flag leaf stage and weighed for the determination of fresh weight (FW). After that, leaf samples were dipped in water and weighed to determine the turgid weight (TW). Later on, turgid leaves were ovendried, and dry weight was taken. Finally, RWC was determined by the following equation given by Mostofa and Fujita (2013).


$$RWC\left(\%\right)=\frac{F\,W-D\,R}{T\,W-D\,R}\times 100$$

Fresh leaves (0.25 g) were added in 25 ml of distal water after 24 h EC_1_ was recorded using an EC meter. Then the test tubes were heated in the water bath for 50 min at 90°C, and EC_2_ was recorded. The final value of electrolyte leakage was calculated by the equation:


E⁢L%=(E⁢C⁢1÷E⁢C⁢2)×100.

### Determination of H_2_O_2_ and Malondialdehyde Levels

Hydrogen peroxide concentration in rice samples was measured at the flag leaf stage by Velikova et al. (2000). A total of 0.5 g rice plant sample was grounded in 5 ml of TCA solution and centrifuged. After that, samples were placed in test tubes, 1M potassium iodide (KI) and 100 μl potassium phosphate buffers were added and left at room temperature for 30 min, and absorbance was noted at 390 nm to determine H_2_O_2_ contents. MDA contents in rice plant samples were estimated by Rao and Sresty’s (2000) procedures. For this, 0.5 g frozen sample of rice plants was homogenized in 5 ml of TCA solution and centrifuged at 12,000 rpm for 15 min. After that, the mixture was added with 5 ml of thiobarbituric acid (TBA), heated for 30 min, later on, cooled quickly, and absorbance was noted at 600 nm.

### Determination of Total Soluble Proteins and Free Amino Acids

The concentration of TSP in the rice plant sample was measured at the flag leaf stage using the methods described by Bradford (1976). A total of 0.5 g of plant samples were grounded in phosphate buffer (5 ml) and centrifuged at 15,000 rpm for 15 min. After that, 1 ml plant extract was taken in test tubes containing 3 ml of Bradford reagent and set aside for 15 min at room temperature. Later on, TSP concentration in collected samples was recorded at 595 nm using a spectrophotometer. The total free amino acids (FAA) in the rice sample at the flag leaf stage were determined with Hamilto and Van-Slyke (1943). We took 1 ml of plant extract and placed it in test tubes containing 1 ml of ninhydrin and pyridine solution, and samples were placed in the water bath at 90°C for 30 min. After that, volume was brought to 25 ml, and FAA concentration was recorded at 570 mM using a spectrophotometer. To determine soluble sugars, plant supernatant was prepared, and 1–2 drops were placed on the prism of a digital refractometer to measure soluble sugar value. Bates et al. (1973) methods were used to determine proline contents in rice samples. A total of 0.5 g of rice samples were extracted with 3% sulfosalicylic acid solution (10 ml) and centrifuged for 10 min at 10,000 rpm. Afterward, acid-ninhydrin was added to the supernatant and placed at 90°C in the water bath for 30 min, and absorbance was recorded at 520 nm to determine proline concentration.

### Antioxidant Enzyme Activity

The activities of all the antioxidants were measured at the flag leaf stage. To determine the ascorbate peroxide (APX), we took plant extract and mixture contained 100 μl of enzyme extracts, 100 μl of ascorbate (7.5 mM), 100 μl of H_2_O_2_ (300 mM), and 2.7 ml of potassium buffer (25 mM); 2 mM CA having 7.0 pH was added in the plant extract. Absorbance was noted at 290 nm with a spectrophotometer to determine APX activity (Nakano and Asada, 1981). After that, APX was determined at 290 nm using the spectrophotometer. The activity of ascorbic acid (AsA) was measured using the procedure described by Mukherjee and Choudhuri (1983). A total of 0.5 g rice samples were standardized with 10% tri-chloro-acetic acid solution (5 ml) and centrifuged at 8,000 rpm for 10 min. Afterward, 2 ml of supernatant was taken, and 0.5 ml DTC reagent was added and incubated for 3 h and then cooled. The procedure given by Aebi (1984) was used to determine the catalase (CAT) contents in rice samples. Test tubes containing 100 μl of H_2_O_2_ (5.9 mM) and 1,000 μl of buffer along with the 100 μl of plant extract were taken and centrifuged at 15,000 rpm for 30 min, and absorbance was noted at 240 nm using a spectrophotometer. The peroxidase (POD) content was measured by the procedure of Zhang (1982). We took reactants having 100 μl of extract enzyme + 2,700 μl of 50 mM potassium buffers + 100 μL guaiacol and 100 μl of H_2_O_2_, which was added into plant sample. Afterward, rice samples were homogenized with 5 ml of potassium phosphate buffer (50 mM having 7.0 pH) under ice-cold conditions and centrifuged for 15 min at 15,000 rpm. Absorbance was recorded using a spectrophotometer at 470 nm.

### Determination of Elemental Concentration

Rice plant samples were taken and washed to remove any contamination. After that, sample were ovendried and grinded to make the powder. Finally, grinded samples were digested at a hot plate after adding the mixture of HCL and HNO_3_, and the filtrate was obtained and diluted by adding water. The concentration of ions (Na^+^ and K^+^) in rice was measured using a flame photometer (Jones and Case, 1990).

### Determination of Yield Attributes

The tillers of all the plants in every pot were counted and averaged to determine the tillers/pot. Similarly, panicles on each plant in every pot were calculated, and the average was taken. Five panicles from each pot were selected, panicle lengths were measured, and grains/panicles were counted. The full pots were harvested, and grains were separated from the panicle and weighed to determine the grain yield/pot.

### Statistical Analysis

The observed data were analyzed with computer-based software STATISTIX 8.1 using analysis of variance, and the least significant differences (LSD) test at 0.05 probability level was used to determine the significant difference among means (Steel et al., 1997). Moreover, Sigma-plot (8.0) software was used to prepare the graphs.

## Results

### Effect of Press-Mud Amendment on Growth Attributes of Rice Plants Grown Under Salinity Stress

The results indicated that salinity markedly reduced the growth-related parameters of rice (Table 1). Shoot and root length decreased significantly (0.05 P) by 37.5 and 24.1% at 6 and 12 dS m^–1^, respectively (Table 1). Likewise, salt stress also diminished biomass production. The maximum reduction in roots fresh weight (16.7%), shoots fresh weight (35.6%), root dry mass (22.2%), and shoot dry mass (37.4%) at 12 dS m^–1^ was compared with control (Table 1). However, PM application protecting the rice plants from harmful impacts of salinity stress significantly improved the growth traits. Application of PM (9%) increased shoot and root length by 44.7 and 27.9%, shoot and root fresh biomass by 40.7 and 7%, and shoot and root dry biomass by 41.5 and 23.9%, respectively, under 12 dS m^–1^ salinity stress (Table 1).

**TABLE 1:** Effect of different levels of press mud application on growth attributes of rice plants grown under different levels of salinity stress.

SL	PM L	SL (cm)	RL (cm)	SFW (g)	RFW (g)	SDW (g)	RDW (g)	LPP
0 dS m^–1^	Control	49.7e ± 0.9	11.8bc ± 0.7	55.0g ± 0.8	3.7cd ± 0.04	10.4ef ± 0.7	1.4e ± 0.02	23f ± 0.80
	3%	66.0d ± 0.8	14.7a ± 0.9	71.0d ± 0.6	3.8c ± 0.03	13.9c ± 0.8	1.5d ± 0.01	28cd ± 0.8
	6%	75.7b ± 10	14.9a ± 0.8	80b ± 1.30	3.9b ± 0.05	15b ± 1.30	1.7b ± 0.02	31b ± 0.70
	9%	80.0a ± 1.3	15.6a ± 1.0	85a ± 1.40	3.9a ± 0.15	16.1a ± 0.8	1.8a ± 0.04	33a ± 0.80
3 dS m^–1^	Control	35.1j ± 0.8	9.7e ± 0.70	41.0j ± 0.8	3.4fg ± 0.01	6.7i ± 0.9	1.1h ± 0.03	18.5h ± 1.2
	3%	41.2i ± 1.2	10.8cd ± 0.4	44.0i ± 0.7	3.5f ± 0.02	9.1g ± 0.3	1.3f ± 0.03	20.2g ± 1.2
	6%	61.8e ± 0.9	12.8b ± 0.6	66.0e ± 0.8	3.6e ± 0.02	12.5d ± 0.5	1.5d ± 0.01	27d ± 0.90
	9%	71.0c ± 0.8	14.8a ± 0.8	74.7c ± 1.0	3.7d ± 0.03	14c ± 0.40	1.6c ± 0.01	29c ± 0.60
6 dS m^–1^	Control	29.0k ± 0.6	9.2e ± 0.80	35.1k ± 0.6	3.0k ± 0.08	5.5j ± 1.0	1.1i ± 0.03	17.5h ± 0.8
	3%	41.0i ± 0.9	10.1de ± 0.8	41.2j ± 1.1	3.2j ± 0.03	8.5h ± 0.9	1.2g ± 0.02	20g ± 0.40
	6%	44.0h ± 0.7	11.7bc ± 0.7	49.7h ± 0.9	3.3i ± 0.03	9.5g ± 1.1	1.3f ± 0.04	21g ± 0.60
	9%	55.0f ± 0.4	12.2b ± 0.9	61.5f ± 1.1	3.4h ± 0.02	11e ± 0.80	1.4e ± 0.02	25.5e ± 0.7
LSD at 0.05 P		0.746	0.533	0.744	0.042	0.739	0.020	0.659

*SL, salinity levels; PML, press mud levels; SL, shoot length; RL, root length; SFW, shoot fresh weight; RFW, root fresh weight; SDW, shoot dry weight; RDW, root dry weight; LPP, leaves per plant. The values given in the table are the mean of three replicates with ± S.E. and different letters with each meaning showing the significant difference at p ≤ 0.05.*

### Effect of Press-Mud Amendment on Photosynthetic Pigments of Rice Plants Grown Under Salinity Stress

**FIGURE 1:**
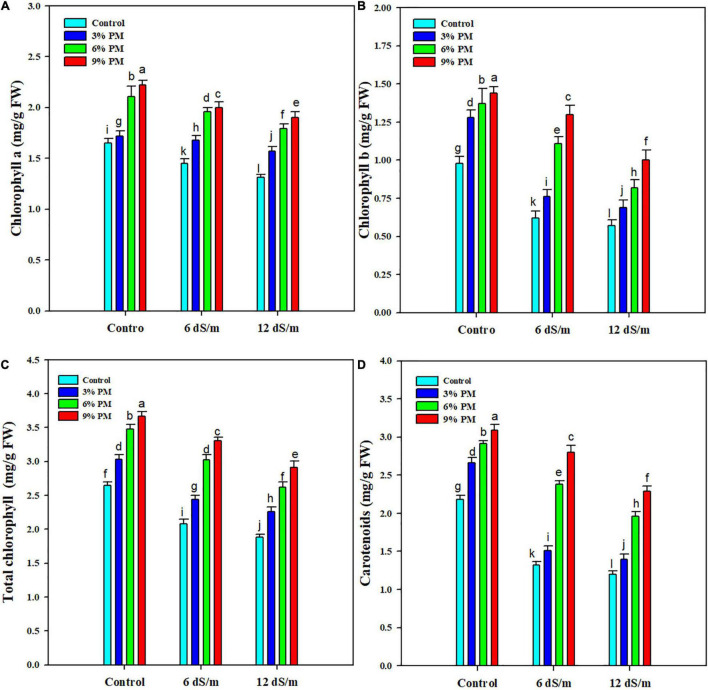
Effect of different levels of press mud application on chlorophyll **(A)**, chlorophyll **(B)**, total chlorophyll **(C)**, and carotenoid **(D)** contents of rice crop grown under different levels of salinity stress. The bars indicate the means of three replications with ± S.E. and a different letter indicating significant differences at *p* < 0.05.

Salt stress significantly (*p* = 0.05) reduced chlorophyll and carotenoid contents in rice seedlings (Figure 1). The chlorophyll a, chlorophyll b, total chlorophyll, and carotenoids showed a reduction of 15.1, 39.4, 25, and 37.2% under a salinity stress level of 12 dS m^–1^ (Figure 1). The PM application significantly increased photosynthetic pigments under salinity stress (Figure 1). An escalation in chlorophyll a (28%), chlorophyll b (41.6%), total chlorophyll (33%), and carotenoid (42.4%) was recorded with PM (9%) under salt stress as compared with control (Figure 1).

### Effect of Press-Mud Amendment on Electrolyte Leakage and Relative Water Contents of Rice Plants Grown Under Salinity Stress

**FIGURE 2:**
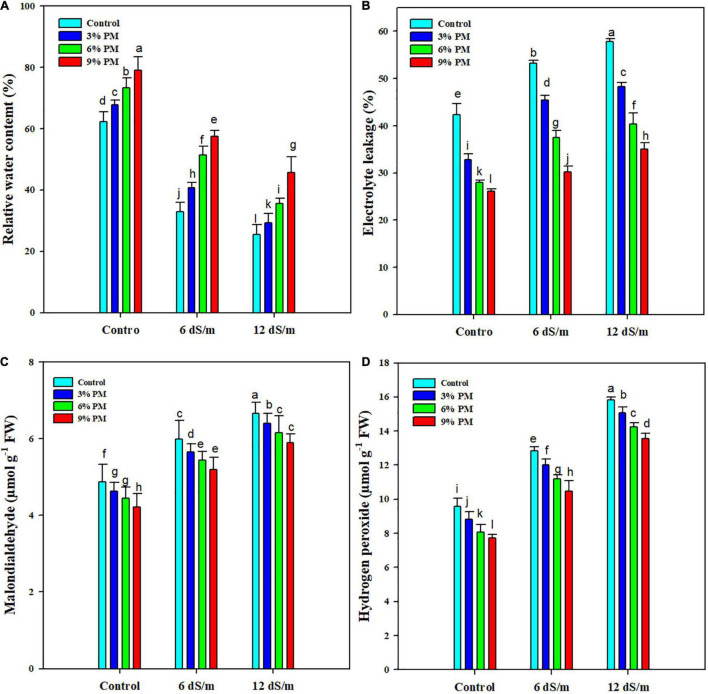
Effect of different levels of press mud application on RWC **(A)**, EL **(B)**, MDA **(C)**, and H_2_O_2_ **(D)** contents of rice crop grown under different levels of salinity stress. The bars indicate the means of three replications with ± S.E. and a different letter indicating significant differences at *p* < 0.05.

The PM application markedly reduced the EL and considerably increased the RWC (Figure 2). Maximum EL (37.2%) and minimum RCW (50.6%) were recorded at 12 dS m^–1^ salt stress compared with control. Moreover, PM application (9%) reduced electrolyte leakage by 42% and increased RWC by 34% under salt stress (12 dS m^–1^) conditions as compared with control (Figure 2).

### Effect of Press-Mud Amendment on Malondialdehyde and H_2_O_2_ Contents of Rice Plants Grown Under Salinity Stress

Salt stress significantly increased the MDA accumulation, and maximum MDA contents (6.55 μmol g^–1^ FW) were recorded in 12 dS m^–1^ salt stress without PM application while the lowest MDA contents (4.2 μmol g^–1^ FW) was noted in control with 9% PM application (Figure 2). The application of salinity stress and PM also significantly affected H_2_O_2_ contents (Figure 2). The maximum concentration of H_2_O_2_ (15.82 μmol) was observed under 12 dS m^–1^ salinity stress without PM application, and the minimum concentration of H_2_O_2_ (7.73 μmol) was noticed under control conditions. The application of PM significantly reduced H_2_O_2_ accumulation; however, application of PM (9%) remained the top-performing, and it reduced the H_2_O_2_ accumulation by 14 and 17% and at 6 and 12 dS m^–1^, respectively (Figure 2).

### Effect of Press-Mud Amendment on Total Soluble Protein, Free Amino Acids, Soluble Sugars, and Proline Content of Rice Plants Grown Under Salinity Stress

**FIGURE 3:**
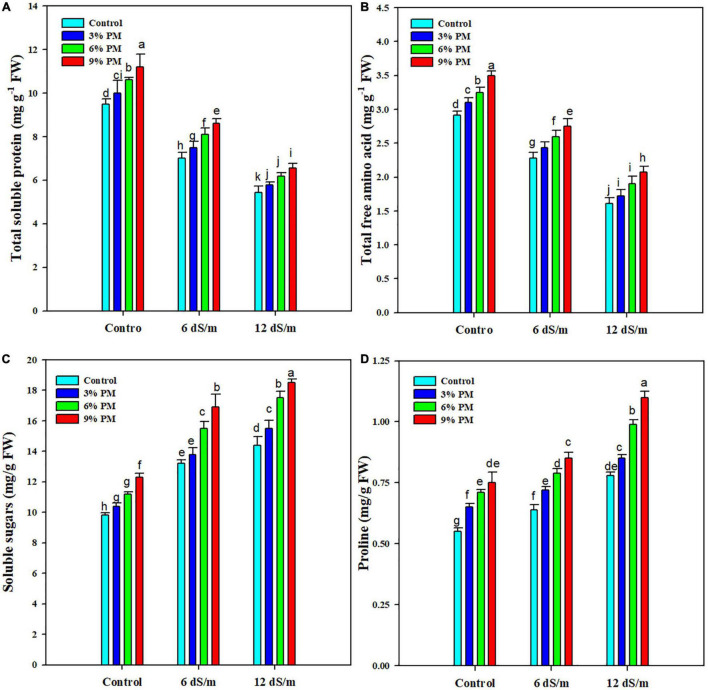
Effect of different levels of press mud application on TSP **(A)**, FAA **(B)**, soluble sugars **(C)**, and proline **(D)** contents of rice crop grown under different levels of salinity stress. The bars indicate the means of three replications with ± S.E. and a different letter indicating significant differences at *p* < 0.05.

Salinity stress considerably reduced TSP and FAA concentrations (Figure 3), and a reduction of 38 and 39% in TSP and FAA, respectively, was recorded at 12 ds m^–1^ salt stress (Figure 3). However, PM appreciably increased the accumulation of both TSP and FAA. The application of PM (9%) increased the TSP and FAA by 18 and 19%, respectively, at 12 dS m^–1^ salt stress (Figure 3). The results indicated that soluble sugars (SS) and proline contents were significantly increased under salt stress (Figure 3). Further application of PM also increased the accumulation of SS and proline (Figure 3). The PM application (9%) increased SS by 27 and 28% at 6 and 12 dS m^–1^ salt stress, respectively, while it increased the proline contents by 33 and 41% at 6 and 12 dS m^–1^ salt stress, respectively, as compared with control (Figure 3).

### Effect of Press-Mud Amendment on the Activity of Antioxidant Enzymes of Rice Plants Grown Under Salinity Stress

**FIGURE 4:**
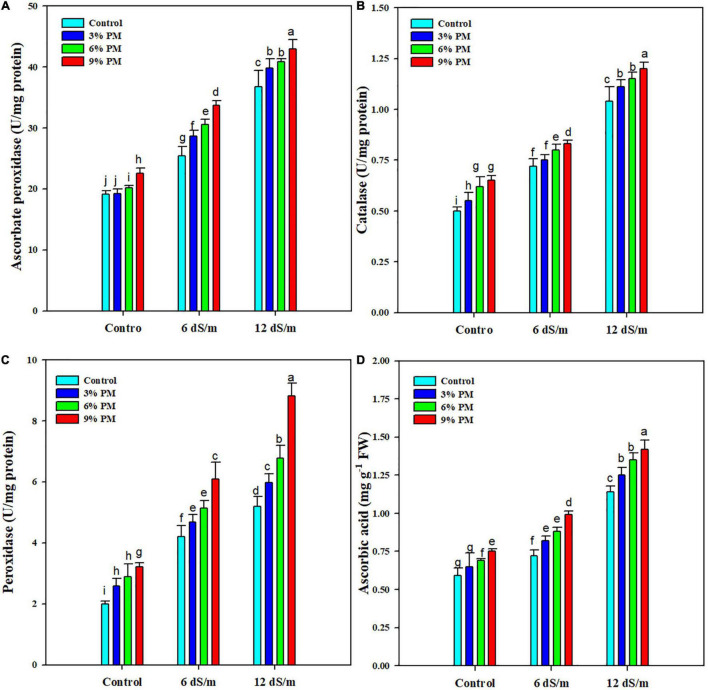
Effect of different levels of press mud application on APX **(A)**, CTA **(B)**, POD **(C)**, and AsA **(D)** contents of rice crop grown under different levels of salinity stress. The bars indicate the means of three replications with ± S.E. and a different letter indicating significant differences at *p* < 0.05.

Results revealed that salt stress and PM application considerably increased the antioxidant enzyme activities (Figure 4). The PM (9%) increased activities of CAT (12 and 15.8%) and APX (10 and 13%) under both levels of salt stress (Figure 4). Likewise, POD and AsA activities also increased by 52 and 38% with PM (9%) under a salt stress level of 12 dS m^–1^ compared with control treatment (Figure 4).

### Effect of Press-Mud Amendment on the Elemental Concentration of Rice Plants Grown Under Salinity Stress

**FIGURE 5:**
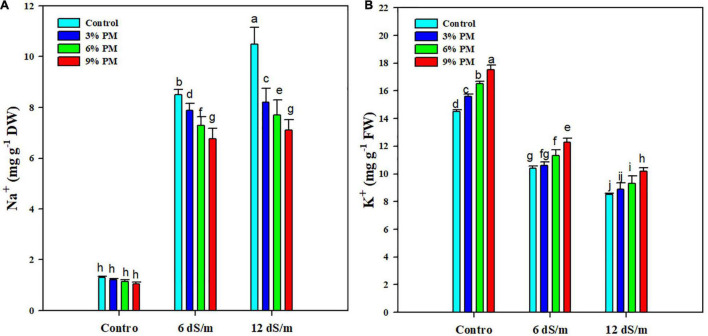
Effect of different levels of press mud application on Na^+^ **(A)**, and K^+^ **(B)** contents of rice crop grown under varying levels of salinity stress. The bars indicate the means of three replications with ± S.E. and a different letter indicating significant differences at *p* < 0.05.

Salt stress significantly increased the Na^+^ contents while it reduced the K^+^ accumulation. Conversely, PM appreciably reduced the Na^+^ while increasing K^+^ accumulation (Figure 5). The application of a PM (9%) reduced the Na^+^ contents by 26 and 48% under moderate (6 dS m^–1^) and stronger (12 dS m^–1^) salt stress levels (Figure 5). Moreover, the application of PM (9%) increased the K^+^ contents by 18 and 20% at moderate (6 dS m^–1^) and stronger (12 dS m^–1^) salt stress (Figure 5).

### Effect of Press-Mud Amendment on Yield and Yield Parameters of Rice Plants Grown Under Salinity Stress

Salinity caused a significant decrease in yield attributes of rice crops (Table 2). However, PM appreciably improved the yield traits of rice (Table 2). The maximum tillers (8.3), panicle length (20.6 cm), and panicle/plant (12.5) were noted in control (no salt stress) with the application of 9% PM, whereas the lowest tillers (3.8), panicle length (8 cm), and panicle/plant (6.5) were recorded at 12 dS m^–1^ without PM application (Table 2). Likewise, PM application also markedly increased the grains/panicle, thousand-grain weight (TGW), and grain yield/pot under normal and salt stress conditions. The application of 9% PM remained at the top position, and it significantly improved the grains/panicle (195 and 198%), TGW (21.03 and 23.61%), and grain yield/pot (105 and 86%) at 6 and 12 dS m^–1^ salt stress (Table 2).

**TABLE 2:** Effect of different levels of press mud application on yield and yield attributes of rice plants grown under different levels of salinity stress.

SL	PML	NT	PL (cm)	PPP	GPP	TGW (g)	GY/pot (g)
0 dS m^–1^	Control	5.9e ± 0.6	13.5g ± 0.8	9.4cd ± 1.2	12.0g ± 0.8	18.82de ± 0.59	26.6g ± 0.9
	3%	7.1bc ± 0.8	17.1c ± 0.6	10.5bc ± 0.5	16.5d ± 0.5	19.60cd ± 0.46	34.3d ± 0.8
	6%	8.0a ± 0.8	19.7ab ± 0.4	11.5ab ± 1.2	19.5b ± 0.5	21.30b ± 0.78	39.5b ± 0.6
	9%	8.3a ± 0.6	20.6a ± 1.1	12.5a ± 1.1	21.0a ± 0.7	23.33a ± 0.85	42.5a ± 0.9
3 dS m^–1^	Control	4.2i ± 0.7	8.9j ± 0.01	7.3ef ± 1.1	6.1k ± 0.9	16.83g ± 0.58	18.0j ± 0.6
	3%	5.0fg ± 0.8	11.1i ± 0.2	8.6de ± 1.2	9.0i ± 0.8	18.00ef ± 0.46	22.5i ± 2.4
	6%	6.6c ± 0.4	16.0cd ± 0.7	10.6bc ± 0.4	15.1e ± 0.6	19.80cd ± 0.53	32.4e ± 1.7
	9%	7.6b ± 0.4	18.2c ± 0.8	11.5ab ± 0.9	18bc ± 0.9	20.37bc ± 0.96	37.4c ± 1.6
6 dS m^–1^	Control	3.8j ± 0.1	8.0k ± 0.8	6.5f ± 1.0	4.5l ± 0.5	15.67g ± 0.74	15.9k ± 0.7
	3%	4.6h ± 0.9	9.8ij ± 0.5	7.5ef ± 0.8	7.4j ± 0.6	17.23fg ± 0.46	19.3j ± 0.3
	6%	5.4f ± 0.5	12.2gh ± 0.6	8.4de ± 0.6	10.5h ± 0.3	19.43cd ± 0.21	24.4h ± 1.1
	9%	6.3cd ± 0.6	14.9f ± 0.4	9.5cd ± 0.8	13.4f ± 0.7	19.37cd ± 1.00	29.5f ± 1.5
LSD at 0.05 P		0.490	1.014	0.707	0.351	1.14	0.834

*SL, salinity levels; PML, press mud levels; NT, number of tillers; PL, panicle length; PPP, panicle per plant; GPP, grains per panicle; TGW, thousand-grain weight; GY, grain yield. The values given in the table are the mean of three replications with ± S.E. and different letters with each meaning showing the significant difference at p ≤ 0.05.*

## Discussion

Mitigation of salinity stress through eco-friendly approaches is highly demanding in plant production for sustainable agriculture and global food security. In this study, salinity stress decreased rice plants’ growth, biomass production, and photosynthetic efficiency and induced oxidative stress. However, it was evident that the SPM significantly alleviated salinity stress and improved rice growth and yield by improving physiological and biochemical attributes. Salinity stress induced a substantial increase in Na^+^ accumulation, decreasing photosynthetic pigments and leaf water contents disturbed nutrient and water uptake, thereby reducing growth and biomass production (Mahmood et al., 2021; Nahar et al., 2022). However, the PM amendment appreciably alleviated the salinity stress and improved the growth and biomass production. PM application enhances soil organic matter content, which improves the nutrient and water uptake and maintains better synthesis of photosynthetic pigments, resulting in a significant improvement in growth and biomass production (Budiyanto, 2021; Imran et al., 2021). The favorable conditions created by SPM also improved the antioxidant activities and osmolytes activities, which protected the cellular structure, proteins, and lipids from the toxic effect of salinity, and enhanced plant growth and biomass production (Imran et al., 2021; Sheoran et al., 2021a). In this study, salinity stress significantly reduces the photosynthetic pigments. Excessive Na^+^ ions participate in ROS production by working as signaling molecules in transduction pathways (Fatima et al., 2021). The excessive accumulation of Na^+^ denatures the enzyme needed to synthesize chlorophyll and therefore reduces the synthesis of chlorophyll (Alzahib et al., 2021). However, PM alleviated this reduction, which indicates PM application reduced the excessive Na^+^ accumulation, which protects photosynthetic apparatus from damaging effects of Na^+^ and reduces the activities of chlorophyll degrading enzymes, improving chlorophyll synthesis under salinity stress (Sheoran et al., 2021a). In rice plants, salinity stress significantly reduced RWC (Figure 1). Plants exposed to salinity stress face the osmotic challenge that reduces the water uptake. Besides this, ABA-mediated stomatal closure affects the transpiration pull and leads to low/no water uptake by plant roots and entails low RWC in plants (Meguekam et al., 2021). In contrast, PM improved RWC, which could be due attributed to an increase in cell turgor pressure, water uptake, and reduced transpiration rate, which resulted in a significant increase in RWC (Kumar and Chopra, 2016; Soni et al., 2016).

Salinity significantly increased the accumulation of MDA and H_2_O_2_. The increased H_2_O_2_ accumulation interrupts normal cell functioning by causing oxidative damage and substantially reducing growth and productivity (Hassan et al., 2020; Sultan et al., 2021). Membrane damage is the primary effect of salinity stress, and increased MDA accumulation under salt stress that can be attributed to salt-induced membrane damage (Fatima et al., 2021). Salt stress significantly increased EL; however, the PM amendment markedly reduced the EL. Salt stress increases ROS production, which damages cell membranes and consequently increases the EL (Sultan et al., 2021). Membrane integrity plays an imperious role in salt tolerance, and reduction in EL with PM application was linked with lower MDA and H_2_O_2_ accumulation owing to improved antioxidant activities (APX, CAT, POD, and AsA) and accumulation of proline and soluble sugars (Sheoran et al., 2021a).

Salinity stress significantly reduced TS and FAA while it increased the accumulation of soluble sugars and proline. The higher FAA accumulation creates a potential osmotic gradient which facilitates inward water movement and prevents the plants from toxic effects of salinity stress (Sultan et al., 2021). PM increases nitrogen uptake, which increases protein synthesis because nitrogen is an integral component of proteins. Another possible reason for this increase in protein concentration might be increased antioxidant activities due to PM, which protected the proteins from damaging effects of salinity stress and improved their accumulation under salinity stress. Increased protein accumulation regulates metabolic processes and antioxidant activities, which enhances salt tolerance (Fahad and Bano, 2012; Farouk and Al-Huqail, 2020). The increase in sugars accumulation improves salt tolerance (Fahad and Bano, 2012), and in this study, the PM amendment significantly increased the accumulation of soluble sugars in rice plants. Proline accumulates in plants in response to salinity stress which confers salt tolerance (Sultan et al., 2021). Proline accumulation was significantly increased under salt stress, and further SPM also increased the proline accumulation. Press mud application increases the activity of the proline synthesis enzyme (pyrroline-5-carboxylate reductase), which increases proline synthesis and accumulation and improves salt tolerance (Kumar A. et al., 2017; Makarana et al., 2019; Sheoran et al., 2021a).

The activities of antioxidant enzymes were increased under salinity stress, which was further increased by PM application. The increase in activities of antioxidants substantially scavenges the ROS and protects the plants from the damaging effects of salt-induced oxidative stress (Parveen et al., 2019; Mahmood et al., 2021). However, the mechanism behind PM-induced increase in antioxidant activities is still unexplored. Therefore, further studies must be conducted to explore the mechanism behind the increased antioxidant activity with PM supplementation. An inadequate K^+^ supply under salt stress reduces the photosynthetic rate and causes oxidative damage, which are primary reasons for a reduction in growth and yield (Hasanuzzaman et al., 2018; Dustgeer et al., 2021). Na^+^, is a toxic ion, interferes with K^+^ uptake, which disturbs the stomatal conductance, water uptake and induces necrosis and water loss, therefore, causing significant loss in growth and yield (Larbi et al., 2020).

Additionally, at the early stages of salinity stress, higher Na^+^ concentration also disturb the Ca^2+^ level and subsequently impair the Ca^2+^ availability to young leaves (Talaat and Shawky, 2022). A particular amount of Ca^2+^ is needed to maintain membrane integrity leaves (Talaat and Shawky, 2022); therefore, a reduction in rice growth in the current study can be linked with a decrease in membrane permeability due to poor Ca^2+^ uptake. However, PM appreciably reduced the Na^+^ accumulation in rice plants by increasing the K^+^ accumulation. Press mud application ensures the desirable Ca^2+^ availability in soil by mobilizing CaCO_3_, improving soil structural stability, and increasing leaching of Na^+^ (Prapagar et al., 2012), thereby reducing the salinity-induced oxidative damages (Sheoran et al., 2021b). Additionally, PM being an excellent nutrient source ensures a better supply of K^+^ in salt-affected soils, and Na^+^ uptake minimizes the salt-induced toxic effects (Kumar S. et al., 2017).

Salinity stress significantly reduced the yield and yield contributing traits of rice crops. Increased Na^+^ accretion in rice leaves due to salt stress-induced early leaf senescence, reduced the panicle formation and assimilated production, reducing the growth and yield traits (Mannan et al., 2013). Salt stress also reduces photosynthetic pigments and disrupts osmolytes accumulation, plant water relationships, membrane integrity, and K^+^ uptake and therefore causes a reduction in yield and yield traits (Al-Ashkar et al., 2019; Otie et al., 2021). Press mud alleviated the adverse impacts of salinity stress and improved rice yield and yield traits. Improved soil properties, nutrient uptake, photosynthetic pigments, antioxidant activities, osmolytes accumulation, K^+^ uptake, and reduced MDA and H_2_O_2_ following PM application substantially improved the yield and yield traits.

## Conclusion

Rice development and yield were substantially hampered by salinity stress due to a considerable rise in MDA, H_2_O_2_, Na^+^, and electrolyte leakage. Surprisingly, salinity-induced negative effects were restored mainly due to the application of sugarcane press mud. The application of press mud (9%) significantly improved rice growth and yield due to improved photosynthetic pigment, relative water contents, osmoregulating compounds, and K^+^ accumulation. It reduced MDA, H_2_O_2_, Na^+^, and electrolyte leakage through triggered antioxidant activities. Therefore, the use of press acerbated deleterious impacts of salinity stress provides strong evidence for the role of press mud in improving the salinity tolerance in rice plants. However, more genomics, transcriptomic, proteomics, and metabolomics studies are direly needed to underpin the mechanism associated with press mud-induced salinity tolerance in rice plants.

## Data Availability Statement

The datasets presented in this study can be found in online repositories. The names of the repository/repositories and accession number(s) can be found in the article/supplementary material.

## Author Contributions

IK and MC conceived the idea. AM performed the experiment. IK, MC, and MH prepared the draft of manuscript. MBi, MA, MRi, WS, MS, MAR, MBr, MZ, and AE reviewed and edited the final version. All authors contributed to the article and approved the submitted version.

## Conflict of Interest

The authors declare that the research was conducted in the absence of any commercial or financial relationships that could be construed as a potential conflict of interest.

## Publisher’s Note

All claims expressed in this article are solely those of the authors and do not necessarily represent those of their affiliated organizations, or those of the publisher, the editors and the reviewers. Any product that may be evaluated in this article, or claim that may be made by its manufacturer, is not guaranteed or endorsed by the publisher.
